# Professional health care use and subjective unmet need for social or emotional problems: a cross-sectional survey of the married and divorced population of Flanders

**DOI:** 10.1186/1472-6963-12-420

**Published:** 2012-11-22

**Authors:** Elien Colman, Sara Symoens, Piet Bracke

**Affiliations:** 1 Department of Sociology, Hedera (Health and demographic research), Ghent University, Korte Meer 5, Ghent, 9000, Belgium

**Keywords:** Divorce, Health care use, Subjective unmet need, Perceived unmet need, Mental health

## Abstract

**Background:**

The high mental health care consumption rates of divorced singles may constitute a heavy burden on the public health care system. This raises the question of whether their higher health care use stems from a greater need, or whether there are other factors contributing to these high consumption rates. We examine both health care use and subjective unmet need (perceiving a need for care without seeking it) because of social or emotional problems of the divorced singles, the repartnered divorcees, and the married. Moreover, we investigate how health care use and subjective unmet need relate to each other.

**Methods:**

We conduct several gender specific logistic regressions employing data from the Divorce in Flanders Survey (N men = 2884; N women = 3317).

**Results:**

Results show that the **divorced singles** have more contact with professional health care providers (general practitioners, psychiatrists, and psychologists) because of social or emotional problems, and more often perceive unmet needs. The higher health care use rates and greater subjective unmet needs can largely be attributed to higher levels of depressive symptoms. Surprisingly, we find that non-frequent health care users more often perceive a subjective unmet need than frequent health care users and those who have not contacted any health care provider.

**Conclusion:**

The single divorced consult health care providers more often because of social or emotional problems and they also perceive unmet needs more often*.*

## Background

For decades, research has consistently indicated the benefits of marriage and the detrimental consequences of divorce for mental health [1-8]. Divorced singles, especially, experience higher levels of depression, stress, and fear, as well as lower levels of self-esteem [9-13]. Although remarriage after divorce is beneficial for mental health, the continuously married still have a higher level of well-being when compared to those in higher order marriages [1,5,14]. However, as none of the studies on the determinants of mental health care use has made the distinction between the continuously married and those who are repartnered after divorce, little is known about how these mental health differences translate into differences in health care seeking because of social or emotional problems. Some studies have shown that divorced singles have a higher rate of health care use than the married population, even after their mental or physical health status has been considered [15-19]. This higher level of use places a heavy burden on the public health care system. Hence, questions arise as to whether both the single divorcees and the repartnered divorcees have a higher rate of health care use than the married, and whether disparities can be attributed to a greater need for care or to other factors that may contribute to high consumption rates.

Research on the study of disparities in professional health care use has focused on the prevalence and the determinants of unmet need. Unmet need arises when someone with a health issue does not receive the care that person needs [20-22]. Usually, research is based on measures of need-adjusted health care utilization, in which the mental health care use rates of different social groups are compared after controlling for their mental health status. Therefore, need for care is estimated using standardized scales after controlling for socio-demographic factors. Because this approach has been criticized for its shallowness, some researchers have turned to a more subjective approach, letting respondents themselves assess whether they have experienced a need for care without getting this care. The present study combines both research strategies to get a more complete picture of the prevalence and correlates of unmet need. By looking at current partner status, we aim to reveal differences between the married, the divorced living with a new partner, and divorced singles (a) in their contact with general practitioners (GPs), psychiatrists, or psychologists and (b) in the prevalence of a perceived need for care without seeking that care. Simultaneously, this study examines how these two outcomes relate to each other. By doing so, it makes two contributions: First, it adds to the literature on the consequences of divorce on levels of health care use, thereby examining and determining the impact of new partnerships. Second, we study the interrelatedness of health care use rates and subjective unmet need.

### Conceptual model

The most important theoretical framework on this topic is Andersen’s Behavioral Model of Health Services Use [23], which describes how the level of health care use is determined by predisposing, enabling, and need factors. Predisposing factors are mainly socio-demographic and socio-economic characteristics—gender, age, marital status, number of children in the household, education, and employment—that indicate the likelihood that someone will seek professional care. Enabling factors are those qualities that make it easier to receive care, such as financial resources and social support [23]. In this study, we focus on need for care because of social or emotional problems. The Andersen Behavioral Model is most often applied in studies based on the need-adjusted measurement approach, but can also be considered a useful frame of reference for the study of subjective unmet need [20,24].

#### Consequences of divorce regarding mental health and health care use

Divorce and its possible negative consequences increase stress levels and can result in the deterioration of mental health [1,2,4,6,7,9,12-14,25-27]. Divorced singles in particular have more mental health problems, but those who have repartnered also tend to have somewhat worse mental health than the continuously married [1,5,14]. It has been shown that divorce has detrimental consequences for mental health, particularly in the short term [28], but also, to a lesser extent, in the long term [2,29]. Previous studies have however indicated that the higher levels of health care use of the divorced singles cannot be attributed entirely to their worse mental health status [19,30]. If we assume that these higher levels are the consequence of divorce, it could be anticipated that repartnered divorcees will also have a greater health care use than expected based on their mental health status, compared to their married counterparts. Therefore, we hypothesize the following:

H1a Divorced singles are more likely than their married counterparts to consult a professional health care provider, even after considering need for care.

H1b Divorcees who are currently living with a new partner are more likely to consult a professional health care provider compared to the continuously married, even after considering need for care.

Moreover, research also indicates that those in poor health report more unmet need, suggesting that their need for care is not entirely met [24,31,32]. Based on these findings, we hypothesize the following:

H1a Divorced singles are more likely to perceive an unmet need compared to their married counterparts.

H2b Divorcees who are currently living with a new partner are more likely to perceive an unmet need compared to their married counterparts.

H3 Differences in professional health care use and subjective unmet need between the married and both the repartnered and single divorcees diminish with the elapse of time since the divorce.

As we still expect to find differences in mental health care use based on partner status after considering need for care [19,30], there must be additional factors contributing to these disparities. Because divorce not only affects need factors, enabling factors and predisposing characteristics should also be considered:

#### Enabling factors

The crowding-out hypothesis suggests that social support can substitute for professional care [32,33]. As such, the higher rate of health care use among the divorced singles found in previous research may also be due to the lower amount of social support received by divorcees [25]. In contrast, the crowding-in hypothesis asserts that social support encourages professional care seeking: friends and relatives help to identify mental health problems and motivate people to seek professional care [19,34,35].

Those in a better financial situation have better mental health [36,37]; they can also afford more health care and therefore perceive unmet need less often [24,32,38,39]. Therefore, we can expect the divorced singles to experience more financial barriers to health care, because they are in general economically less well off, particularly women [2,11,40-42].

Most studies on barriers to health care are conducted in the United States, indicating that being insured is an important enabling factor [24,32]. The present study takes place in Flanders, the Dutch-speaking part of Belgium, which has a broadly accessible health care system that covers a wide range of health services. The government largely finances the health care system and it is compulsory to have health insurance. In this context, we would expect to find few disparities and, as a consequence, little subjective unmet need. Nevertheless, research shows that there are still significant disparities in Belgium, especially with respect to specialist care [38].

#### Predisposing characteristics

Women have a higher health care use rate than men do. Some researchers assert that this can be attributed to their worse mental health [30,43], but most studies conclude that the higher health care use rate among women cannot be explained completely by need factors, and postulate that women are in general more inclined to seek professional care [19,44,45]. Nevertheless, research indicates that they also experience more barriers to care [46] and report unmet need more often [20,24,31,47].

Women often have custody of children, which adds demanding care tasks. The impact of the presence of children in the household is often neglected in the literature on health care use and subjective unmet need [30,39,47]. Although some studies find no effect on mental health care use [38], delay of care, or subjective unmet need [31], the presence of children may be important among the divorced. The presence of stepchildren adds to the complexity of the household; having joint children with the ex-partner is also often considered a stressor, with negative effects on the mental health of divorcees [2,48,49].

Aging is associated with worsening somatic and mental health problems and, therefore, a higher rate of health care use [39,43]. Research consistently shows that lower education is a predictor of subjective unmet need [24,32], but there are conflicting findings concerning the association between education and level of health care use [15,19,38]. Moreover, the unemployed, the retired, and the homemaker have less contact with health care providers [19,38,39], although they tend to have more mental health problems [50,51]. Among the employed, those working full time have less contact with a GP for a mental health problem [30]. There are conflicting findings however about the influence of employment on subjective unmet need [24,32].

### Objective and subjective assessments of unmet need

Research on disparities in health care use is usually based on measures of need-adjusted utilization: After controlling for health status, the amount of health care use is compared between social groups. There are three major criticisms of this operationalization. First, research on utilization assumes that those who score high on an indicator of need by definition do need professional care. But some people prefer to deal with these problems on their own by changing lifestyles, turning to cognitive behavioral approaches, or relying on their support networks [47]. For some, these strategies might be effective and, as such, they perceive no need for professional care. Likewise, it is assumed that people who score low on an indicator of mental health problems do not need care. However, some sub-threshold cases would benefit from mental health services in order to prevent more serious problems and might as such perceive a need for care. Second, commonly used measures of need might be inadequate indicators of need for professional health care [21]. Finally, this operationalization does not consider either patient satisfaction or whether the delivered care is appropriate and effective. Consequently, researchers implicitly assume that professional care is helping people who receive it.

These limitations have inspired researchers to turn to a subjective approach: They ask respondents whether they perceive an unmet need for health care. In the present study, we define subjective unmet need as *perceiving a need for care because of social or emotional problems without actually seeking this care*.

By combining both research strategies, we aim to achieve a more extensive understanding of the prevalence of unmet need and its correlates. When combining strategies, the question arises of how measures of need-adjusted health care use and measures of subjective unmet need relate to each other. Although ten Have et al.’s study found that patients who have received care state more often that they would seek care if they were faced with a serious emotional problem again [52], several studies have shown that health care use heightens the chance of subjective unmet need [20,22,47]. Based on these findings, we hypothesize that 

H4: Those who receive professional health care more often perceive an unmet need.

## Methods

### Sample

We employed the Divorce in Flanders Survey (DiF) data (http://www.divorceinflanders.be) [53]. The survey is based on a sample of marriages instead of individuals. More specifically, we got permission from the Commission for the Protection of Privacy (CPP) to take a sample from the Public Register of heterogamous intact and broken marriages conducted in Flanders, legally formed between 1971 and 2008, of which both partners have been Belgian nationals since birth and are currently alive and domiciled in Flanders. Only first marriages were included; broken marriages in which one or both partners have divorced more than once were excluded.

Marriages were selected by proportionally stratified sampling by marriage cohort.^a^ To make sure that the broad diversity of the population of divorcees would be captured, broken marriages were oversampled (one third intact vs. two thirds broken) within every marriage cohort. Both partners (or current ex-partners) were contacted individually for their participation**.** Respondents were questioned during 2009–2010 using a computer-assisted personal interview (CAPI). Response rates were 43.3% among the divorced and 39.5% among the married. There were 1093 ex-couples and 756 married couples of which both partners participated in the survey. As the data of the DIF sample are partially clustered, the assumption of independence of observations is threatened. To avoid inferential errors, gender specific analyses were performed (N men = 2884; N women = 3317). Characteristics of both samples of men and women are presented in Additional file 1.

### Analysis procedure

We carried out gender specific logistic regressions analyses to identify the correlates of health care use and then the correlates of subjective unmet need. First, a baseline model was estimated, introducing only predisposing characteristics (Model 0). Subsequently, enabling (Model 1) and need factors (Model 2) were introduced. In the analysis on subjective unmet need, an extra model (Model 3) was added, introducing an indicator of frequency of care use. Only the results of the full models are presented in the paper; the result of the other models can be found in the Additional files.

### Variables

#### Dependent variables

With regard to health care use, a dichotomous measure was used as several studies based on comparisons with registered data have indicated that measures of *any* medical use (0–1) were more accurate than measures of the quantity of use [54-56]. Respondents who had contacted a GP, psychiatrist, or psychologist because of social or emotional problems in the year before the interview were given a score of 1 (16.4%) on *professional health care use.*

We measured *subjective unmet need* using the question, “Was there ever a time in the past 12 months when you thought you needed professional help for your social or emotional problems, but you did not seek professional help?” Of all respondents, 8.2% answered yes.

#### Predisposing characteristics

With regard to *partner status*, we distinguished the continuously married from the divorced currently living with a new partner and the single divorced. The continuously married were set as reference category. To include the time elapsed since the divorce, two internal product terms were added: Divorced, new partner × years divorced; and divorced, no partner × years divorced (Congruent with Mirowksy [57]).

*Age* was computed based on birth date and date of the interview. We calculated the respondent’s *number of children* under the age of 12 and the number of children between 12 and 21 years old living in the household. Among the divorcees living with a new partner, we also considered the presence of stepchildren. Congruent with Mirowsky [57], internal product terms were added (child of partner < 12 * divorced, new partner; child of partner ≥ 12 * divorced, new partner). All respondents score 0 on these product terms, except those repartnered divorcees who have at least one stepchild living in their household. Three *educational levels* were defined: high (tertiary and non-tertiary), middle (upper secondary; reference category), and low educational level (preprimary, primary, and lower secondary). Concerning *employment status*, we determined three categories: working full time, working part time, or not being employed; working full time was used as a reference category.

#### Enabling factors

*Equivalent household income* (EHI) was calculated as indicated by the Equivalent Income OECD modified scale, and was based on information on household income and received alimony. Five income categories were determined, each separately for the sample of men and the sample of women: EHI missing, less than 50%, 50–79%, 80–119%, and 120% or more of the median EHI. To measure the available *social support,* we calculated the number of people **(with a maximum of five)** one can go to for a personal conversation.

#### Need

*Depression* was adopted as indicator of mental health and measured by the 8-item version of the Center of Epidemiologic Studies Depression (CES-D) scale (Chronbach’s alpha = 0.840). This scale was constructed to identify populations at risk for developing depressive disorders [58]. Because we view depression as a continuum, cutoff points were not applied. Higher scores on the CES-D scale indicate higher levels of the quantity and severity of depressive symptoms.

As *self-rated health* relates to both physical and mental health [59,60], it was also added as an indicator of need for care. It was measured by asking “Would you say your health is…” with answers ranging from very bad (1) to excellent (5).

#### Frequency of health care use

For each health care provider contacted, the respondent was asked how often they had contacted this care provider during the 12 months preceding the interview. Possible answer categories were (1) once, (2) several times a year, (3) monthly, (4) every 2 weeks, and (5) weekly or more. Based on this information, three categories of frequency of health care use were constructed: (1) those who did not visit any health care provider because of social or emotional problems were identified as *non-users*; (2) those who had contacted one or more health care provider once, and those who had contacted one health care provider several times a year were identified as *non-frequent health care users*; and (3) those who had consulted one or more health care providers more often were identified as *frequent health care users*. Two dummy variables were created, using the non-frequent health care users as a reference category.

## Results

### Descriptives

We find that among both men and women the continuously married experience the fewest depressive symptoms and have the highest self-rated health, whereas the divorced singles have the worst score on these indicators of need for care (see Additional file 2). Those with more depressive symptoms and those with worse self-rated health contact help care providers more often and also report a subjective unmet need more often (see Table 1).

**Table 1:** Prevalence of health care use and subjective unmet need among men and women

	**MEN**								**WOMEN**							
	**Health care use**				**Subjective unmet need**				**Health care use**				**Subjective unmet need**			
	**N = 317**				**N = 169**				**N = 699**				**N = 339**			
	**%**		**N**		**%**		**N**		**%**		**N**		**%**		**N**	
Partner status																
Married	7.5		60		3.6		29		14.0		128		7.4		68	
Divorced, new partner	9.2		117		4.2		54		19.1		249		8.5		111	
Divorced, no partner	17.3		140		10.6		86		29.4		322		14.6		160	
Employment status																
Not employed	20.0		87		9.0		39		30.9		216		12.9		90	
Part time	12.2		20		9.8		16		19.9		229		9.1		104	
Full time	9.2		210		5		114		17.3		254		9.9		145	
Education																
Low	13.7		94		5.5		38		26.5		171		9.3		60	
Middle	10.6		125		4.6		54		20.4		275		9.9		134	
High	9.6		98		7.6		77		19.2		253		11.0		145	
Equivalent household income																
< 50% mean EHI	17.0		32		11.2		21		31.2		88		14.9		42	
50-79% mean EHI	14.2		114		6.4		51		25.8		253		12.2		119	
80-119% mean EHI	9.1		90		5.2		52		19.2		194		8.3		84	
≥ 120% mean EHI	9.0		61		5.3		36		16.5		116		9.5		67	
EHI Missing	9.0		20		4.1		9		14.0		48		7.8		27	
	**Health care use**		**No health care use**		**Subjective unmet need**		**No subjective unmet need**		**Health care use**		**No health care use**		**Subjective unmet need**		**No subjective unmet need**	
	**Mean**	**SD**	**Mean**	**SD**	**Mean**	**SD**	**Mean**	**SD**	**Mean**	**SD**	**Mean**	**SD**	**Mean**	**SD**	**Mean**	**SD**
Age	46.69	8.18	47.50	8.09	45.27	8.61	47.55	8.05	45.12	7.89	45.44	8.13	44.63	8.17	45.46	8.07
N children of R < 12	0.46	0.91	0.43	0.79	0.59	1.03	0.42	0.79	0.46	0.80	0.52	0.87	0.55	0.89	0.50	0.85
N children of R ≥ 12	0.31	0.66	0.36	0.71	0.30	0.63	0.36	0.71	0.64	0.87	0.56	0.80	0.60	0.84	0.57	0.82
Social support (0–5)	3.01	1.51	2.58	1.55	2.82	1.51	2.62	1.56	3.49	1.36	3.18	1.44	3.46	1.37	3.22	1.44
Depression (0–24)	8.04	5.24	4.36	2.99	9.16	4.90	4.49	3.21	8.68	5.16	5.06	3.56	9.68	5.24	5.38	3.85
Self-rated health	3.49	0.94	3.95	0.69	3.54	0.83	3.92	0.73	3.45	0.87	3.94	0.68	3.55	0.84	3.87	0.74

Descriptive results show that divorced singles have the highest rates of health care use and are more likely to perceive a need for care without seeking it. Divorced women who are currently living with a new partner also have a somewhat higher health care use rate (19.1%) compared to married women (14.0%). No differences are found between repartnered and continuously married men however with regard to health care use and subjective unmet need.

### Correlates of contact with professional health care providers

The results of all steps of the logistic regressions with regard to contact with a professional health care provider are shown in Additional file 3 for men and in Additional file 4 for women. Results of the final model are shown in Table 2. For both men and women, results reveal that even after considering all predisposing and enabling factors, no differences in health care use between the married and the repartnered divorced are found, while the effect of being single divorced remains. Single divorced men (OR Model 1: 2.279***) and women (OR Model 1: 3.247***) are more likely to have contacted a health care provider because of social or emotional problems than their married counterparts. We find no effect of time since divorce among men, but among single divorced women, the longer the time since the divorce, the less likely they are to contact a professional care provider (OR Model 1: 0.979*).

**Table 2:** Correlates of health care use, considering predisposing, enabling, and need factors (results of logistic regressions)

	**MEN**		**WOMEN**	
	**OR**	**CI**	**OR**	**CI**
Constant	0.089 ***		0.166 ***	
**Partner status** (Ref. cat. = married)				
Divorced, new partner	1.165	0.680 - 1.997	1.272	0.858 - 1.886
Divorced, no partner	1.481	0.922 - 2.380	2.316 ***	1.649 - 3.253
Divorced, new P * years divorced	1.001	0.971 - 1.031	1.001	0.979 - 1.024
Divorced, no P * years divorced	1.010	0.980 - 1.040	0.980	0.958 - 1.002
Age	0.979 *	0.960 - 0.999	0.972 ***	0.957 - 0.988
N children of R < 12	1.224 *	1.029 - 1.455	0.891	0.775 - 1.024
N children of R ≥ 12	1.007	0.822 - 1.234	1.081	0.962 - 1.214
N stepchildren < 12 * new partner	0.964	0.633 - 1.468	0.932	0.565 - 1.536
N stepchildren ≥ 12 * new partner	0.915	0.615 - 1.361	1.073	0.678 - 1.699
**EHI** (Ref. Cat. = 80-120% mean)				
EHI < 50%	1.266	0.765 - 2.093	1.113	0.775 - 1.598
EHI 50-80%	1.225	0.877 - 1.712	0.971	0.759 - 1.242
EHI 120%+	1.136	0.779 - 1.657	0.945	0.713 - 1.253
EHI missing	0.737	0.420 - 1.295	0.674 *	0.464 - 0.980
Social support	1.244 ***	1.142 - 1.356	1.281 ***	1.193 - 1.375
**Education** (Ref. cat. = middle)				
Low	1.215	0.882 - 1.674	1.347 *	1.041 - 1.743
High	0.872	0.632 - 1.203	1.138	0.909 - 1.425
**Employment status** (Ref. cat. = full-time work)				
Part-time work	1.215	0.703 - 2.099	1.314 *	1.055 - 1.636
Not employed	1.734 **	1.202 - 2.502	1.726 ***	1.311 - 2.271
Depression	1.205 ***	1.164 - 1.247	1.163 ***	1.137 - 1.190
Self-rated health	0.784 **	0.656 - 0.936	0.652 ***	0.568 - 0.748
Nagelkerke R^2^	20.8		23.7	
Log Likelihood	1681.5		2868.3	

*p < 0.05; **p < 0.01; ***p < 0.001.

These higher health care use rates among single divorcees can largely be attributed to their higher need for care (see Table 2): After adding depressive symptoms and self-rated health into the analysis, the effect of being single divorced is no longer statistically significant among men (OR 1.481) and is remarkably lower among women (OR 2.316***). Moreover, the higher likelihood of health care use among the more recently divorced single women can also to a large extent be ascribed to their higher need for care, as the effect of years since divorce is no longer significant only after need factors are considered (OR 0.980).

With regard to these need factors, it holds that for both men and women there is a greater likelihood of contacting a health care provider for those who report more depressive symptoms (OR men: 1.205***; OR women: 1.163***) and who score worse on self-rated health (OR men: 0.784**; OR women 0.652***).

Concerning the other predisposing characteristics, the odds that an individual will contact a professional health care provider are greater for the young (OR men: 0.979*; OR women: 0.972***), the unemployed (OR men 1.734***; OR women 1.726***), for part-time working women (OR 1.314*), and less educated women (OR 1.347*), and for men with small children (OR 1.281*). Among women, we find no effects of children in the household.

With regard to the enabling factors, we find no association with EHI, except that among women, those who have a missing value on the income measure are less likely to have consulted a health care provider (OR 0.674*). Among both men and women, the results show a positive association with social support (OR men: 1.244***; OR women: 1.281***). This is in line with the crowding-in hypothesis; the more persons one can rely on for a personal conversation, the more likely one is to seek professional care.

### Correlates of subjective unmet need

When controlling for predisposing and enabling characteristics (see Model 2 in Additional file 5 for men, Additional file 6 for women), we find no differences in subjective unmet need between married and divorced men who are currently living with a new partner. Repartnered women are however more likely than married women to experience a need for care without seeking it (OR 1.802*). This association becomes weaker with the increase of time elapsed since the divorce (OR 0.971*). After introducing depressive symptoms and self-rated health (see Model 3, Additional file 5) into the analysis, the effect of being a repartnered divorced woman is no longer significant, indicating that the higher prevalence of subjective unmet need among divorced women without a new partner can be attributed to their worse mental health status.

Moreover, when considering predisposing and enabling factors (see Model 2 in Additional file 5 for men, Additional file 6 for women), both single divorced men (OR 4.548***) and women (OR 2.886***) have a much higher risk of perceiving need without actually seeking care compared to their continuously married counterparts. After controlling for depressive symptoms and self-rated health (see Model 3 in Additional file 5 for men, Additional file 6 for women), the odds of perceiving a need for care without seeking it remain high among single divorced men (OR 2.769***) and women (OR 1.923*). Among women, this association diminishes with the elapse of time since the divorce (OR 0.965*).

With regard to the indicators of need, we find that depressive symptoms are positively related to the perception of an unmet need, whereas self-rated health is not associated with the presence of a subjective unmet need. Results also indicate that, among both men and women, available social support is not related to perceiving a need without seeking care.

For men, findings (see Model 1 in Additional file 5) indicate that the odds of experiencing unmet need are greater among the young (OR 0.951***), the highly educated (OR 1.918**), those not working full time (OR part-time work: 2.732; OR not employed: 2.514***), those in the lowest income category (OR 1.934*), and those having more small children living in the household (OR 1.145**). After considering depressive symptoms and self-rated health, only the effects of not being poor and unemployed disappear (see Model 2 in Additional file 5 for men). The young (OR 0.957***, Model 2 in Additional file 5), the highly educated (OR 2.029***), and men with young children (OR 1.388) are more likely to perceive an unmet need than we would expect based on their need for care.

Among women (see Model 1, Additional file 6), we also find that those in the lowest income groups (OR 1.662*) and those working part time (OR 1.013**) are more likely to perceive an unmet need. These findings can be attributed to their worse mental and self-rated health (see Model 2, Additional file 6). Among women, age, number of (step)children, education, and not being employed are not associated with perceiving a need for professional care without seeking care.

### The association between contact with a health care provider and subjective unmet need

To examine the interrelatedness of health care use and subjective unmet need, frequency of health care use is introduced in the final step of the analysis (Table 3, Model 4). Results show that non-frequent health care users are more likely than non-users to perceive a need for care without seeking this care (OR No health care use, men: 0.306***; OR No health care use, women: 0.276***). Among women, frequent health care users are less likely to perceive an unmet need (OR 0.600*).

**Table 3:** Correlates of subjective unmet need considering predisposing, enabling, and need factors and frequency of care use (results of logistic regressions)

	**MEN**		**WOMEN**	
	**OR**	**CI**	**OR**	**CI**
Constant	0.111 *		0.061 **	
**Partner status** (Ref. cat. = married)				
Divorced, new partner	1.453	0.677 - 3.120	1.501	0.876 - 2.572
Divorced, no partner	2.575 **	1.392 - 4.764	1.653 *	1.064 - 2.569
Divorced, new P * years divorced	0.986	0.948 - 1.026	0.977	0.949 - 1.007
Divorced, no P * years divorced	0.989	0.945 - 1.035	0.967	0.935 - 1.001
Age	0.958 **	0.932 - 0.984	0.991	0.971 - 1.012
N children of R < 12	1.334 **	1.076 - 1.654	1.060	0.888 - 1.266
N children of R ≥ 12	0.998	0.757 - 1.315	0.968	0.829 - 1.130
N stepchildren < 12 * new partner	1.184	0.692 - 2.028	1.369	0.796 - 2.352
N stepchildren ≥ 12 * new partner	1.081	0.645 - 1.813	1.157	0.658 - 2.034
**EHI** (Ref. cat. = 80-120% mean)				
EHI < 50%	1.707	0.902 - 3.231	1.381	0.856 - 2.228
EHI 50-80%	0.894	0.559 - 1.430	1.138	0.814 - 1.591
EHI 120%+	1.041	0.636 - 1.705	1.193	0.819 - 1.737
EHI missing	0.601	0.267 - 1.353	1.090	0.666 - 1.786
Social support	0.986	0.875 - 1.110	1.098	1.000 - 1.205
**Education** (Ref. cat. = middle)				
Low	1.039	0.641 - 1.681	0.787	0.546 - 1.135
High	2.117 ***	1.378 - 3.251	1.280	0.956 - 1.715
**Working situation** (Ref. cat. = fulltime work)				
Part-time work	2.251 *	1.175 - 4.313	0.906	0.675 - 1.216
Not employed	1.555	0.917 - 2.638	1.046	0.723 - 1.513
Depression	1.240 ***	1.185 - 1.296	1.182 ***	1.148 - 1.217
Self-rated health	1.019	0.801 - 1.297	1.035	0.863 - 1.240
Frequency of health care use (Ref. cat. = non frequent)				
No health care use	0.306 ***	0.191 - 0.489	0.276 ***	0.205 - 0.370
Frequent health care use	0.535	0.274 - 1.046	0.600 *	0.403 - 0.894
Nagelkerke R^2^	26.1		22.6	
Log Likelihood	1002.3		1804.6	

*p < 0.05; **p < 0.01; ***p < 0.001.

## Discussion

When interpreting the results, it is important to keep some limitations in mind. First, the Divorce in Flanders Survey is a cross-sectional survey in which respondents were asked whether they had contacted a professional health care provider during the past year and whether they had felt a need for care but did not seek professional help during the past year. Because we consider self-rated general health and experiencing depressive symptoms during the week preceding the interview to be predictors of professional health care use and subjective unmet need during the last year, caution is needed when making causal interpretations. Nonetheless, the finding that among divorced singles the odds of using health care and perceiving an unmet need decrease with the elapse of time since the divorce suggests a causal effect of divorce that diminishes over time. The cross-sectional design of the survey also hinders a causal interpretation of the association between health care use and subjective unmet need. However, because we explicitly defined subjective unmet need as perceiving a need without seeking professional help, it seems reasonable to assume that among those who have reported both professional care use and a subjective unmet need, the professional care use either precedes the subjective unmet need or is based on other complaints. Nevertheless, more research based on longitudinal data is required to be able to make causal conclusions.

Second, the indicators of need for care, depressive symptoms, and self-rated health might not capture all of the reasons people might contact a GP, psychiatrist, or psychologist because of social or emotional problems. Yet because we control for depressive symptoms, we do consider the most common mental health problem in Europe [61]. Moreover, self-rated health is widely used as an indicator of need because it has a good prognostic value [62], even for mental health [63,64].

Third, as lay people often experience mental health problems as somatic symptoms [65], a considerable number of visits to health care providers concerning somatic problems that are in fact symptoms of mental health problems are not included when only health care use because of social or emotional problems is considered.

Fourth, we cannot generalize our results to the whole population, because never-married people, widowed people, and people with multiple divorces are excluded and the divorced are overrepresented. Because this study’s focus is on the still-growing group of the divorced, who are substantial consumers of health care, the Divorce in Flanders Survey is well suited to our purposes because it includes large number of divorcees and detailed information on both marital history and health care use.

Because of growing medical costs, financial resources for public health care in Belgium are strained, as they are in most other Western countries. Moreover, the high consumption rates of the growing category of single divorcees place a heavy burden on the public health care system. This raises the question of whether this high level of health care use is equitable. Results show that both health care use and subjective unmet need because of emotional or social problems are strongly associated with being single after divorce.

In line with hypothesis 1a, we find that the divorced singles are more likely to contact a professional health care provider. This seems a consequence of the lack of a partner rather than of the divorce itself, as the repartnered divorced and the continuously married are comparable regarding their health care use. Among men, we find no differences between the continuously married and the repartnered divorced. Among women, we find that the divorced currently living with a new partner are somewhat more likely to have contacted a professional health care provider, but this can be completely attributed to their worse mental health. Hence, hypothesis 1b cannot be confirmed. As divorced singles have the worst performance with regard to various health-related behaviors, like smoking, alcohol intake, physical activity, eating habits, treatment adherence, and so on [66-71], it is remarkable that, in accordance with other studies [15-19], we find higher rates of health care use than we would expect based on their need for care. A possible explanation may be that these divorced singles have sought help from a professional care provider with regard to problems that most other people can discuss with their partner, or with regard to problems arising from the stress that stems from having the sole responsibility of maintaining the household.

With regard to the prevalence of subjective unmet need, a similar pattern occurs. Disparities between the married and the divorced living without a new partner are pronounced, whereas differences between repartnered divorcees and their married counterparts are less clear cut. Again, no differences are found between repartnered divorced men and their married counterparts. Repartnered women however are somewhat more likely to experience a need for care for which they do not seek professional help. Hence, hypothesis 2b can be confirmed only among women. Congruent with hypothesis 2a, we find that single divorced men and women are much more likely to experience a need for care without seeking this care. Even after considering all predisposing characteristics, enabling factors, depressive symptoms and self-rated health, these disparities remain. This is a remarkable finding, as it has been well illustrated that divorced singles are a vulnerable group, experiencing social and economic disadvantages, which results in higher rates of mental health problems. Apparently, however, this does not completely explain their higher rates of perceived unmet need.

Time elapsed since the divorce seems to matter only among women. However, when considering need for care, the time effect on health care use disappears. Hence, it can be assumed that this negative time effect on health care use reflects the amelioration of mental health with the elapse of time since divorce.

Depressive symptoms are an important correlate of health care use and subjective unmet need. Surprisingly, self-rated health is related to only health care use and not to perceiving a need for care without seeking it. This finding, together with the finding that differences in subjective unmet need between divorced singles and their married counterparts remain after controlling for need for care, shows that the indicator of perceived unmet need captures a need for care as perceived by the respondent that is not related to need indicators such as depressive symptoms and self-rated health. It has been shown that although lay people are well able to estimate their health status [72], their assessment of their own need for care differs significantly from assessments based on standardized diagnostic scales [73]. Hence, we argue that it is important to combine both research strategies. Using the subjective approach, we can identify those who perceive a need but do not seek this care. Furthermore, it is important to determine which individuals recognize a need for professional care but fail to seek it, and to examine why they do not seek this care. Research based on the need-adjusted approach helps to identify which groups health care underrepresents.

We find that those who have had non-frequent contact with a health care provider are the most likely to report a need for care without seeking this care. This is of interest because it challenges the assumption of research based on need-adjusted measures of health care use that the needs of people receiving professional health care are being met, and it raises questions about how these high rates of subjective unmet need among health care users can be explained. At a time when concern about cost-effectiveness in health care is increasing, it is important to determine who these people are that so often perceive a need for care without seeking it, and why they fail to do so. A study based on the ESEMeD (European Study on the Epidemiology of Mental Disorders) data has shown that 19% of current and 30% of former mental health care users think that professional help is as bad, or worse, than no help [52]. One possible explanation for the higher rates of subjective unmet need among non-frequent health care users therefore might be that they are not satisfied with the care received. This dissatisfaction might also be the reason why these non-frequent health care users did not seek professional help more frequently.

We find no apparent effect of income. Nevertheless, we cannot conclude that there are no financial barriers to health care in Flanders, because we did not make a distinction between specialized and non-specialized care. Research has indicated that the poor are more likely to consult non-specialized care providers such as a GP, while those who are financially better off consume more specialized care.

Concerning social support, we find that people who can count on numerous friends and relatives are more likely to contact a health care provider. This finding is in line with the crowding-in hypothesis: Intimates help a person gain insight into personal and emotional problems and encourage a person to seek professional help [19,34,35,74]. But among women we find that those who can count on numerous intimates are also more likely to perceive a need for care without seeking it. This finding is concordant with the crowding-out hypothesis, as it indicates that women who rely strongly on social networks when they need someone to talk to are more reluctant to seek professional care when perceiving a need. Hence, there are indications that both a crowding-in and a crowding-out process are at work among women.

## Conclusion

Despite its limitations, this study provides some interesting insights into the differences between the divorced and the married population’s health care use rate and into the factors that contribute to consumption rates. Single divorced men and women are more likely to consult a health care provider and to perceive an unmet need because of social or emotional problems. This can largely be attributed to their worse mental health. Furthermore, we find that those who have contacted a professional health care provider are more likely to perceive an unmet need than those who had frequent contact with a health care provider and those who have had no contact at all.

By exploring determinants of both health care use rates and perceived unmet need, we offer a multifaceted approach for research on disparities in levels of health care use. Nonetheless, longitudinal research on this topic is needed, especially to determine why health care users so often perceive the need for care without seeking this care.

### Endnotes

^a^In order to account for the proportional stratification by marriage cohort, we conducted extra analyses controlling for marriage cohort. We found no significant differences by marriage cohort concerning health care use and subjective unmet need. Except for the disappearance of the age effects, results were similar to those presented in this paper.

## Abbreviations

DiF: Divorce in Flanders; GP: General practitioner; SHARE: Survey of Health and Retirement in Europe; OR: Odds ratio; CI: Confidence interval; ESEMeD: European Study on the Epidemiology of Mental Disorders; R: Respondent; EHI: Equivalent household income; Ref. cat.: Reference category; Sig: Significance level.

## Competing interests

The authors declare that they have no competing interests.

## Authors’ contributions

EC drafted the manuscript, and analyzed and interpreted the data. SS and PB assisted in data-analyzing interpretation, contributed to writing, and provided feedback on drafts. All authors read and approved the final manuscript.

## Pre-publication history

The pre-publication history for this paper can be accessed here:

http://www.biomedcentral.com/1472-6963/12/420/prepub

## Supplementary Material

Additional file 1 **Characteristics of the sample of men and the sample of women.** Characteristics of the sample of men and the sample of women. Click here for file

Additional file 2 **Mean scores for men and women on depression and self-rated health by partner status.** Mean scores for men and women on depression and self-rated health by partner status. Click here for file

Additional file 3 **Correlates of health care use, considering predisposing (Model 0), enabling (Model 1), and need factors (Model 2) among men (results of logistic regressions).** Correlates of health care use among men. Click here for file

Additional file 4 **Correlates of health care use, considering predisposing (Model 0), enabling (Model 1), and need factors (Model 2) among women (results of logistic regressions).** Correlates of health care use among women. Click here for file

Additional file 5 **Correlates of subjective unmet need considering predisposing (Model 0), enabling (Model 1), and need factors (Model 2) and frequency of care use (Model 3) among men (results of logistic regressions).** Correlates of subjective unmet need among men. Click here for file

Additional file 6 **Correlates of subjective unmet need considering predisposing (Model 0), enabling (Model 1), and need factors (Model 2) and frequency of care use (Model 3) among women (results of logistic regressions.** Correlates of subjective unmet need among women. Click here for file
