# Supplementary material for: Professional health care use and subjective unmet need for social or emotional problems: a cross-sectional survey of the married and divorced population of Flanders
Source: BMC Health Serv Res. 2012 Nov 22;12:420. doi: 10.1186/1472-6963-12-420 (PMC3562142; doi:10.1186/1472-6963-12-420)
Supplement: Additional file 1 — Characteristics of the sample of men and the sample of women. Characteristics of the sample of men and the sample of women. [file 1472-6963-12-420-S1.docx]

**Additional File 1: Characteristics of the sample of men and the sample of women**

|  | **MEN** | | | | **WOMEN** | | | |
| --- | --- | --- | --- | --- | --- | --- | --- | --- |
|  | N = 2884 | | | | N = 3317 | | | |
|  | % | | N | | % | | N | |
| **Health care use** | 11.0 | | 317 | | 21.1 | | 699 | |
| **Subjective unmet need** | 5.9 | | 169 | | 10.0 | | 339 | |
| **Partner status** |  |  |  |  |  |  |  |  |
| Married | 27.6 | | 796 | | 27.6 | | 917 | |
| Divorced, new partner | 28.1 | | 811 | | 33.1 | | 1097 | |
| Divorced, no parter | 44.3 | | 1277 | | 39.3 | | 1303 | |
| **Employment status** |  |  |  |  |  |  |  | |
| Not employed | 15.0 | | 434 | | 21.1 | | 700 | |
| Part time | 5.7 | | 164 | | 34.6 | | 1149 | |
| Full time | 79.3 | | 2286 | | 44.3 | | 1468 | |
| **Education** |  |  |  |  |  |  |  | |
| Low | 23.8 | | 685 | | 19.6 | | 645 | |
| Middle | 40.9 | | 1180 | | 40.7 | | 1351 | |
| High | 35.3 | | 1019 | | 39.8 | | 1321 | |
| **Equivalent household income** |  |  |  |  |  |  |  |  |
| < 50% mean EHI | 6.5 | | 188 | | 8.5 | | 282 | |
| 50-79% mean EHI | 27.8 | | 803 | | 29.5 | | 979 | |
| 80-119% mean EHI | 34.5 | | 994 | | 30.4 | | 1010 | |
| ≥ 120% mean EHI | 23.5 | | 677 | | 21.2 | | 702 | |
| EHI Missing | 7.7 | | 222 | | 10.4 | | 344 | |
|  | **Mean** | | **S.E.** | | **Mean** | | **S.E.** | |
| **Age** | 47.41 | | 8.10 | | 45.38 | | 8.08 | |
| **N children of R < 12** | 0.43 | | 0.81 | | 0.51 | | 0.86 | |
| **N children of R ≥ 12** | 0.36 | | 0.70 | | 0.57 | | 0.82 | |
| **Social support (0-5)** | 2.63 | | 1.56 | | 3.24 | | 1.43 | |
| **Depression (0-24)** | 4.77 | | 3.50 | | 5.82 | | 4.22 | |
| **Self-rated health (1-5)** | 3.90 | | 0.74 | | 3.84 | | 0.75 | |
