# Supplementary material for: Professional health care use and subjective unmet need for social or emotional problems: a cross-sectional survey of the married and divorced population of Flanders
Source: BMC Health Serv Res. 2012 Nov 22;12:420. doi: 10.1186/1472-6963-12-420 (PMC3562142; doi:10.1186/1472-6963-12-420)
Supplement: Additional file 2 — Mean scores for men and women on depression and self-rated health by partner status. Mean scores for men and women on depression and self-rated health by partner status. [file 1472-6963-12-420-S2.doc]

**Additional file 2: Mean scores for men and women on depression and self-rated health by partner status**

|  | **MEN** | | **WOMEN** | |
| --- | --- | --- | --- | --- |
|  | **Depression (0-24)** | | | |
|  | **Mean** | **SE** | **Mean** | **SE** |
| Married | 3.17 | 2.85 | 3.95 | 3.49 |
| Divorced, new partner | 3.21 | 2.99 | 4.36 | 3.99 |
| Divorced, no partner | 5.22 | 4.33 | 6.09 | 4.73 |
|  | **Self-rated health (1-5)** | | | |
|  | **Mean** | **SE** | **Mean** | **SE** |
| Married | 3.99 | 0.64 | 3.98 | 0.66 |
| Divorced, new partner | 3.90 | 0.73 | 3.83 | 0.76 |
| Divorced, no partner | 3.80 | 0.83 | 3.72 | 0.80 |
