# Supplementary material for: Professional health care use and subjective unmet need for social or emotional problems: a cross-sectional survey of the married and divorced population of Flanders
Source: BMC Health Serv Res. 2012 Nov 22;12:420. doi: 10.1186/1472-6963-12-420 (PMC3562142; doi:10.1186/1472-6963-12-420)
Supplement: Additional file 3 — Correlates of health care use, considering predisposing (Model 0), enabling (Model 1), and need factors (Model 2) among men (results of logistic regressions). Correlates of health care use among men. [file 1472-6963-12-420-S3.doc]

**Additional File 3: Correlates of health care use, considering predisposing (Model 0), enabling (Model 1), and need factors (Model 2) among men (results of logistic regressions)**

|  | **Model 0** | | | | **Model 1** | | | | **Model 2** | | |  | |
| --- | --- | --- | --- | --- | --- | --- | --- | --- | --- | --- | --- | --- | --- |
|  | **OR** | | **CI** | | **OR** | | **CI** | | **OR** | | **CI** | | |
| **Constant** | 0.184 | *** |  |  | 0.120 | *** |  |  | 0.089 | *** |  | |  |
| **Partner status** (Ref. cat = married) | |  |  |  |  |  |  |  |  |  |  | |  |
| Divorced, new partner | 1.109 |  | 0.666 - | 1,846 | 1.064 |  | 0.635 - | 1.784 | 1.165 |  | 0.680 - | | 1.997 |
| Divorced, no partner | 2.380 | *** | 1.545 - | 3,667 | 2.279 | *** | 1.463 - | 3.551 | 1.481 |  | 0.922 - | | 2.380 |
| Divorced, new P* years divorced | 1.013 |  | 0.985 - | 1,041 | 1.013 |  | 0.985 - | 1.042 | 1.001 |  | 0.971 - | | 1.031 |
| Divorced, no P * years divorced | 1.017 |  | 0.990 - | 1,045 | 1.010 |  | 0.983 - | 1.039 | 1.010 |  | 0.980 - | | 1.040 |
| **Age** | 0.981 | * | 0.964 - | 0,999 | 0.970 | *** | 0.952 - | 0.988 | 0.979 | * | 0.960 - | | 0.999 |
| **N children of R<12** | 1.136 |  | 0.968 - | 1,333 | 1.162 |  | 0.983 - | 1.373 | 1.224 | * | 1.029 - | | 1.455 |
| **N children of R≥12** | 0.951 |  | 0.791 - | 1,144 | 0.974 |  | 0.803 - | 1.182 | 1.007 |  | 0.822 - | | 1.234 |
| **N stepchildren <12 * new partner** | 0.965 |  | 0.645 - | 1,445 | 0.903 |  | 0.600 - | 1.361 | 0.964 |  | 0.633 - | | 1.468 |
| **N stepchildren ≥12 * new partner** | 0.892 |  | 0.612 - | 1,300 | 0.896 |  | 0.612 - | 1.312 | 0.915 |  | 0.615 - | | 1.361 |
| **EHI** (Ref. cat = 80-120% mean) |  |  |  |  |  |  |  |  |  |  |  | |  |
| EHI <50% |  |  |  |  | 1.531 |  | 0.949 - | 2.469 | 1.266 |  | 0.765 - | | 2.093 |
| EHI 50-80% |  |  |  |  | 1.374 | * | 1.000 - | 1.887 | 1.225 |  | 0.877 - | | 1.712 |
| EHI 120%+ |  |  |  |  | 1.186 |  | 0.826 - | 1.702 | 1.136 |  | 0.779 - | | 1.657 |
| EHI missing |  |  |  |  | 0.936 |  | 0.553 - | 1.584 | 0.737 |  | 0.420 - | | 1.295 |
| **Social support REC** |  |  |  |  | 1.243 | *** | 1.147 - | 1.348 | 1.244 | *** | 1.142 - | | 1.356 |
| **Education** (Ref. cat. = middle) | |  |  |  |  |  |  |  |  |  |  | |  |
| Low |  |  |  |  | 1.245 |  | 0.920 - | 1.684 | 1.215 |  | 0.882 - | | 1.674 |
| High |  |  |  |  | 0.895 |  | 0.660 - | 1.213 | 0.872 |  | 0.632 - | | 1.203 |
| **Employment status** (Ref.cat. = fulltime work) | |  |  |  |  |  |  |  |  |  |  | |  |
| Parttime work |  |  |  |  | 1.548 |  | 0.931 - | 2.574 | 1.215 |  | 0.703 - | | 2.099 |
| Not employed |  |  |  |  | 2.695 | *** | 1.928 - | 3.767 | 1.734 | ** | 1.202 - | | 2.502 |
| **Depression** |  |  |  |  |  |  |  |  | 1.205 | *** | 1.164 - | | 1.247 |
| **Self-rated health** |  |  |  |  |  |  |  |  | 0.784 | ** | 0.656 - | | 0.936 |
| **Nagelkerke R²** | 3.4 | | | | 9.2 | | | | 20.8 | | | | |
| **Log Likelihood** | 1948.6 | | | | 1861.7 | | | | 1681.5 | | | | |

*p < 0.05; **p < 0.01; ***p < 0.001.
