# Supplementary material for: Professional health care use and subjective unmet need for social or emotional problems: a cross-sectional survey of the married and divorced population of Flanders
Source: BMC Health Serv Res. 2012 Nov 22;12:420. doi: 10.1186/1472-6963-12-420 (PMC3562142; doi:10.1186/1472-6963-12-420)
Supplement: Additional file 4 — Correlates of health care use, considering predisposing (Model 0), enabling (Model 1), and need factors (Model 2) among women (results of logistic regressions). Correlates of health care use among women. [file 1472-6963-12-420-S4.doc]

**Additional File 4: Correlates of health care use, considering predisposing (Model 0), enabling (Model 1), and need factors (Model 2) among women (results of logistic regressions)**

|  | | **Model 0** | | | | | **Model 1** | | | | **Model 2** | | | |
| --- | --- | --- | --- | --- | --- | --- | --- | --- | --- | --- | --- | --- | --- | --- |
|  | | **OR** | | | **CI** | | **OR** | | **CI** | | **OR** | | **CI** | |
| **Constant** | | 0,325 | | ** |  |  | 0,305 | *** |  |  | 0,166 | *** |  |  |
| **Partner status** (Ref. cat = married) | |  | |  |  |  |  |  |  |  |  |  |  |  |
| Divorced, new partner | | 1,343 | |  | 0,931 - | 1,938 | 1,427 |  | 0,983 - | 2,072 | 1,272 |  | 0,858 - | 1,886 |
| Divorced, no partner | | 3,079 | | *** | 2,273 - | 4,171 | 3,247 | *** | 2,359 - | 4,468 | 2,316 | *** | 1,649 - | 3,253 |
| Divorced, new P* years divorced | | 1,006 | |  | 0,985 - | 1,027 | 1,002 |  | 0,981 - | 1,023 | 1,001 |  | 0,979 - | 1,024 |
| Divorced, no P * years divorced | | 0,983 | |  | 0,963 - | 1,003 | 0,979 | * | 0,959 - | 0,999 | 0,980 |  | 0,958 - | 1,002 |
| **Age** | | 0,985 | | * | 0,971 - | 0,999 | 0,974 | *** | 0,959 - | 0,988 | 0,972 | *** | 0,957 - | 0,988 |
| **N children of R<12** | | 0,904 | |  | 0,796 - | 1,026 | 0,855 | * | 0,750 - | 0,975 | 0,891 |  | 0,775 - | 1,024 |
| **N children of R≥12** | | 1,066 | |  | 0,961 - | 1,184 | 1,085 |  | 0,973 - | 1,209 | 1,081 |  | 0,962 - | 1,214 |
| **N stepchildren <12 * new partner** | | 0,943 | |  | 0,599 - | 1,486 | 0,935 |  | 0,591 - | 1,479 | 0,932 |  | 0,565 - | 1,536 |
| **N stepchildren ≥12 * new partner** | | 1,016 | |  | 0,668 - | 1,544 | 1,063 |  | 0,694 - | 1,629 | 1,073 |  | 0,678 - | 1,699 |
| **EHI** (Ref. cat = 80-120% mean) | | | |  |  |  |  |  |  |  |  |  |  |  |
| EHI <50% |  | | |  |  |  | 1,282 |  | 0,921 - | 1,785 | 1,113 |  | 0,775 - | 1,598 |
| EHI 50-80% |  | | |  |  |  | 1,143 |  | 0,908 - | 1,439 | 0,971 |  | 0,759 - | 1,242 |
| EHI 120%+ |  | | |  |  |  | 0,991 |  | 0,758 - | 1,296 | 0,945 |  | 0,713 - | 1,253 |
| EHI missing |  | | |  |  |  | 0,729 |  | 0,512 - | 1,038 | 0,674 | * | 0,464 - | 0,980 |
| **Social support** |  | | |  |  |  | 1,249 | *** | 1,168 - | 1,334 | 1,281 | *** | 1,193 - | 1,375 |
| **Education** (Ref. cat. = middle) | | | |  |  |  |  |  |  |  |  |  |  |  |
| Low |  | | |  |  |  | 1,439 | ** | 1,135 - | 1,825 | 1,347 | * | 1,041 - | 1,743 |
| High |  | | |  |  |  | 1,033 |  | 0,836 - | 1,276 | 1,138 |  | 0,909 - | 1,425 |
| **Employment status** (Ref.cat. = fulltime work) | | | | |  |  |  |  |  |  |  |  |  |  |
| Parttime work |  | |  | |  |  | 1,352 | ** | 1,097 - | 1,667 | 1,314 | * | 1,055 - | 1,636 |
| Not employed |  | |  | |  |  | 2,597 | *** | 2,028 - | 3,324 | 1,726 | *** | 1,311 - | 2,271 |
| **Depression** |  | |  | |  |  |  |  |  |  | 1,163 | *** | 1,137 - | 1,190 |
| **Self-rated health** |  | |  | |  |  |  |  |  |  | 0,652 | *** | 0,568 - | 0,748 |
| **Nagelkerke R²** | 4,1 | | | | | | 10,1 | | | | 23,7 | | | |
| **Log Likelihood** | 3326,9 | | | | | | 3193,4 | | | | 2868,3 | | | |

*p < 0.05; **p < 0.01; ***p < 0.001.
