# Supplementary material for: Professional health care use and subjective unmet need for social or emotional problems: a cross-sectional survey of the married and divorced population of Flanders
Source: BMC Health Serv Res. 2012 Nov 22;12:420. doi: 10.1186/1472-6963-12-420 (PMC3562142; doi:10.1186/1472-6963-12-420)
Supplement: Additional file 6 — Correlates of subjective unmet need considering predisposing (Model 0), enabling (Model 1), and need factors (Model 2) and frequency of care use (Model 3) among women (results of logistic regressions. Correlates of subjective unmet need among women. [file 1472-6963-12-420-S6.doc]

**Additional File 6:** **Correlates of subjective unmet need considering predisposing (Model 0), enabling (Model 1), and need factors (Model 2) and frequency of care use (Model 3) among women (results of logistic regressions)**

|  | **Model 0** | | | | | | | **Model 1** | | | | **Model 2** | | | | **Model 3** | | | |
| --- | --- | --- | --- | --- | --- | --- | --- | --- | --- | --- | --- | --- | --- | --- | --- | --- | --- | --- | --- |
|  | **OR** | | | | **CI** | | | **OR** | | **CI** | | **OR** | | **CI** | | **OR** | | **CI** | |
| **Constant** | 0.096 | | *** | |  | |  | 0.059 | *** |  |  | 0.025 | *** |  |  | 0.061 | ** |  |  |
| **Partner status** (Ref. cat = married) |  | |  | |  | |  |  |  |  |  |  |  |  |  |  |  |  |  |
| Divorced, new partner | 1.733 | | * | | 1.050 - | | 2.862 | 1.802 | * | 1.088 - | 2.984 | 1.600 |  | 0.941 - | 2.719 | 1.501 |  | 0.876 - | 2.572 |
| Divorced, no partner | 2.890 | | *** | | 1.951 - | | 4.281 | 2.886 | *** | 1.920 - | 4.340 | 1.923 | * | 1.247 - | 2.966 | 1.653 | * | 1.064 - | 2.569 |
| Divorced, new P* years divorced | 0.973 | | * | | 0.947 - | | 1.000 | 0.971 | * | 0.945 - | 0.998 | 0.973 |  | 0.945 - | 1.002 | 0.977 |  | 0.949 - | 1.007 |
| Divorced, no P * years divorced | 0.965 | | * | | 0.935 - | | 0.997 | 0.965 | * | 0.935 - | 0.997 | 0.965 | * | 0.933 - | 0.999 | 0.967 |  | 0.935 - | 1.001 |
| **Age** | 0.995 | |  | | 0.977 - | | 1.014 | 0.989 |  | 0.970 - | 1.008 | 0.987 |  | 0.967 - | 1.007 | 0.991 |  | 0.971 - | 1.012 |
| **N children of R<12** | 1.055 | |  | | 0.899 - | | 1.238 | 1.012 |  | 0.859 - | 1.192 | 1.040 |  | 0.874 - | 1.237 | 1.060 |  | 0.888 - | 1.266 |
| **N children of R≥12** | 1.014 | |  | | 0.880 - | | 1.168 | 1.018 |  | 0.881 - | 1.176 | 0.987 |  | 0.847 - | 1.150 | 0.968 |  | 0.829 - | 1.130 |
| **N stepchildren <12 * new partner** | 1.368 | |  | | 0.862 - | | 2.172 | 1.354 |  | 0.848 - | 2.163 | 1.371 |  | 0.823 - | 2.285 | 1.369 |  | 0.796 - | 2.352 |
| **N stepchildren ≥12 * new partner** | 1.124 | |  | | 0.669 - | | 1.890 | 1.132 |  | 0.672 - | 1.909 | 1.162 |  | 0.668 - | 2.023 | 1.157 |  | 0.658 - | 2.034 |
| **EHI** (Ref. cat = 80-120% mean) |  | |  | |  | |  |  |  |  |  |  |  |  |  |  |  |  |  |
| EHI <50% |  | |  | |  | |  | 1.662 | * | 1.076 - | 2.569 | 1.423 |  | 0.886 - | 2.283 | 1.381 |  | 0.856 - | 2.228 |
| EHI 50-80% |  | |  | |  | |  | 1.331 |  | 0.973 - | 1.821 | 1.133 |  | 0.814 - | 1.577 | 1.138 |  | 0.814 - | 1.591 |
| EHI 120%+ |  | |  | |  | |  | 1.276 |  | 0.897 - | 1.816 | 1.171 |  | 0.810 - | 1.692 | 1.193 |  | 0.819 - | 1.737 |
| EHI missing |  | |  | |  | |  | 1.071 |  | 0.675 - | 1.699 | 1.052 |  | 0.650 - | 1.701 | 1.090 |  | 0.666 - | 1.786 |
| **Social support** |  | |  | |  | |  | 1.127 | ** | 1.034 - | 1.229 | 1.148 | ** | 1.048 - | 1.257 | 1.098 |  | 1.000 - | 1.205 |
| **Education** (Ref. cat. = middle) |  | |  | |  | |  |  |  |  |  |  |  |  |  |  |  |  |  |
| Low |  | |  | |  | |  | 0.924 |  | 0.660 - | 1.294 | 0.817 |  | 0.569 - | 1.173 | 0.787 |  | 0.546 - | 1.135 |
| High |  | |  | |  | |  | 1.167 |  | 0.889 - | 1.532 | 1.330 |  | 0.998 - | 1.772 | 1.280 |  | 0.956 - | 1.715 |
| **Employment status** (Ref.cat. = fulltime work) | | | | |  | |  |  |  |  |  |  |  |  |  |  |  |  |  |
| Parttime work | |  | |  |  | |  | 1.013 | ** | 0.769 - | 1.335 | 0.974 |  | 0.730 - | 1.298 | 0.906 |  | 0.675 - | 1.216 |
| Not employed | |  | |  |  | |  | 1.653 |  | 1.197 - | 2.283 | 1.125 |  | 0.784 - | 1.614 | 1.046 |  | 0.723 - | 1.513 |
| **Depression** | |  | |  |  | |  |  |  |  |  | 1.210 | *** | 1.177 - | 1.244 | 1.182 | *** | 1.148 - | 1.217 |
| **Self-rated health** | |  | |  |  | |  |  |  |  |  | 0.965 |  | 0.810 - | 1.150 | 1.035 |  | 0.863 - | 1.240 |
| **Frequency of Health care use (ref. cat= non frequent)** | | | | | | |  |  |  |  |  |  |  |  |  |  |  |  |  |
| No | |  | |  | |  |  |  |  |  |  |  |  |  |  | 0.276 | *** | 0.205 - | 0.370 |
| Frequent | |  | |  | |  |  |  |  |  |  |  |  |  |  | 0.600 | * | 0.403 - | 0.894 |
| **Nagelkerke R²** | | 3.2 | | | | | | 5.0 | | | | 18.6 | | | | 22.6 | | | |
| **Log Likelihood** | | 2136.5 | | | | | | 2107.2 | | | | 1876.1 | | | | 1804.6 | | | |

*p < 0.05; **p < 0.01; ***p < 0.001.
